# Spinodal Decomposition and Mechanical Response of a TiZrNbTa High-Entropy Alloy

**DOI:** 10.3390/ma12213508

**Published:** 2019-10-25

**Authors:** Tai-You Liu, Jacob C. Huang, Wen-Shuo Chuang, Hung-Sheng Chou, Jui-Yu Wei, Chih-Yeh Chao, Yu-Chin Liao, Jason S.C. Jang

**Affiliations:** 1 Department of Materials and Optoelectronic Science, National Sun Yat-Sen University, Kaohsiung 804, Taiwan; j199412383@gmail.com (T.-Y.L.); egg037105218@yahoo.com.tw (W.-S.C.); sapphirerei@gmail.com (H.-S.C.); yo8135@gmail.com (J.-Y.W.); 2 Institute for Advanced Study, Department of Materials Science & Engineering, City University of Hong Kong, Kowloon, Hong Kong, China; 3 Department of Mechanical Engineering, National Pingtung University of Science and Technology, Pingtung 912, Taiwan; cychao@mail.npust.edu.tw; 4 Department of Mechanical Engineering, National Central University, Taoyuan 320, Taiwan; llllurker@gmail.com (Y.-C.L.);; 5 Institute of Materials Science and Engineering, National Central University, Taoyuan 320, Taiwan

**Keywords:** high entropy alloy, spinodal decomposition, strengthening, precipitate

## Abstract

In this study, the effects of spinodal decomposition on the microstructures and mechanical properties of a TiZrNbTa alloy are investigated. The as-cast TiZrNbTa alloy possesses dual phases of TiZr-rich inter-dendrite (ID) and NbTa-rich dendrite (DR) domains, both of which have a body-centered cubic (BCC) structure. In the DRs of the as-cast alloy, the α and ω precipitates are found to be uniformly distributed. After homogenization at 1100 °C for 24 h followed by water quenching, spinodal decomposition occurs and an interconnected structure with a wavelength of 20 nm is formed. The α and ω precipitates remained in the structure. Such a fine spinodal structure strengthens the alloy effectively. Detailed strengthening calculations were conducted in order to estimate the strengthening contributions from the α and ω precipitates, as well as the spinodal decomposition microstructure.

## 1. Introduction

In the past decade, high-entropy alloys (HEAs) have become promising materials because of their new concepts of alloy design [1,2,3,4,5]. Unlike conventional alloys, which are based on one major element, HEAs comprise multiple elements in equiatomic or near-equiatomic ratios, and tend to form a simple solid solution instead of complex phases or intermetallic compounds [6,7]. Such a concept opens up new possibilities for advanced alloy design.

High-entropy alloys for high-temperature applications, designed on the basis of Ti, Zr, Nb, Mo, Hf, Ta, and W [8,9,10,11,12], have recently been explored. These alloys present a high compressive strength and acceptable plasticity, mostly a result of their body-centered cubic (BCC) structure [13,14,15]. Recently, researchers have paid attention to the TiZrNbTaMo high-entropy alloys [16,17]. Wang et al. [16] proposed a TiZrNbTaMo HEA possessing a high stiffness and compressive strength, accompanied by an excellent bio-corrosion resistance in the simulated physiological medium. However, the elastic modulus of TiZrNbTaMo HEA for bio-implant materials is 153 GPa, which is much higher than that of human cortical bones (5–40 GPa) [17]. The obvious discrepancy in the elastic modulus would induce a “stress shielding effect” [18,19]. Under such a circumstance, the load would only be carried by the implant, eventually degrading the human bone. Recently, the microstructure and compression properties of quaternary equiatomic TiZrNbTa without Mo addition have been studied by Nguyen et al. [20], in order to compare them with the performance of the conventional biomaterial Ti-6Al-4V. The TiZrNbTa alloy exhibits a compressive plasticity of 48%, which is four times higher than that of Ti-Zr-Nb-Ta-Mo alloys, with a modulus of 116 GPa, similar to Ti-6Al-4V [20]. It should be noted that all four of these elements—Ti, Zr, Nb, and Ta—have been studied for a long time and are classified as highly bio-friendly metals. There is no typical unwanted element, such as Al, Ni, or Cu, in this new category of bio-implant materials.

**Figure 1:**
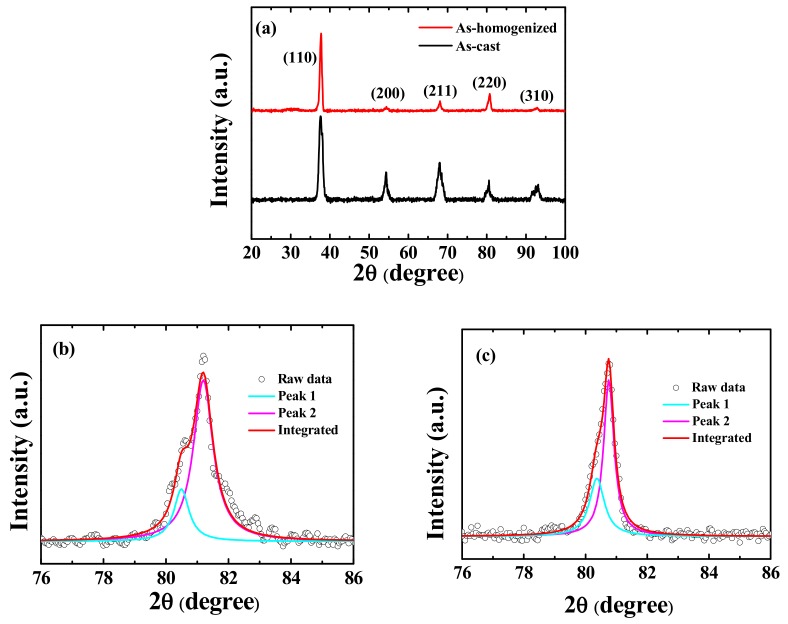
(**a**) The X-ray diffractometry (XRD) analysis of the as-cast and as-homogenized Ti_25_Zr_25_Nb_25_Ta_25_ alloys, (**b**) the enlargement of the (220) peak profile and the fitting curves for body-centered cubic (BCC)1 and BCC2 of the as-cast sample, and (**c**) the enlargement of the (220) peak profile.

If we clearly examine the elements involved in the TiZrNbTa HEA alloy, it is interesting to find that there is basically no or nil heat of mixing (ΔH_m_) between Ti and Zr, as well as between Nb and Ta (both pairs with ΔH_m_ ~0 kJ/mol, as listed in Table 1). This indicates that there are two isomorphous binary phase diagrams for (i) Ti and Zr and (ii) Nb and Ta [21,22], as shown in Figure 1. In other words, Ti and Zr, as well as Nb and Ta, can be classified as twin brothers with similar atomic and physical properties. By mixing these two alloy systems together—TiZr and NbTa—the TiZr-NbTa HEA is formed. In addition to the isomorphous nature of TiZr and NbTa themselves, the heats of mixing between TiNb, TiTa, ZrNb, and ZrTa are in fact all positive [23], as is also listed in Table 1. Due to the positive heat of mixing, a miscibility gap can form over the appropriate composition and temperature range, suggesting the formation of spinodal decomposition. Liang et al. have demonstrated that the initial yield stress of solution-treated Al_0.5_Cr_0.9_FeNi_2.5_V_0.2_ alloy can be increased from 274 to 1810 MPa through the formation of spinodal structures and L1_2_ nano-precipitates [24]. The fine-scaled spinodal microstructure is expected to provide an upgraded strength without raising the elastic modulus [25].

In this research, we intend to explore the development of this new category of bio-friendly TiZrNbTa metallic material. Its interesting spinodal decomposition that occurs in the TiZrNbTa HEAs under the as-cast and as-homogenized conditions is also examined.

## 2. Materials and Methods

TiZrNbTa (i.e., Ti_25_Zr_25_Nb_25_Ta_25_ in at%) samples were prepared from pure metals with a purity higher than 99.9 wt.% by arc melting. To ensure compositional homogeneity, the ingots were re-melted at least five times. Based on the calculated phase diagram of (TiZr)-(NbTa), the ingots of the as-cast alloys were subsequently subjected to homogenization at 1100 °C for 24 h, followed by water quenching, to characterize the spinodal decomposition in the miscibility gap. The as-cast and as-homogenized samples were sectioned, ground, and finally polished sequentially in order to conduct further microstructure analyses.

The crystalline structures were characterized via X-ray diffractometry (XRD) using a Siemens D5000 diffractometer (Karshrule, Germany) with monochromatic CuKα1 + 2 radiation operating at 40 kV/30 mA and a scanning step of 0.05° from 20° to 100°. The microstructures and compositions of the TiZrNbTa samples were characterized via a field-emission-gun scanning electron microscope (SEM, JEOL JSM-6330, Tokyo, Japan) and emission electron probe micro-analyzer (EPMA, JXA-8530F, Tokyo, Japan), respectively. For microstructure analysis via transmission electron microscopy (TEM), the specimens were prepared using the dual-beam focused-ion-beam (FIB) system (Seiko, SMI3050, Tokyo, Japan) with an operating voltage of 30 kV and an ion beam current of 1 pA for the final refinement. The detailed microstructures were characterized by field-emission TEM (Tecnai G20, FEI, Hillsboro, OR, USA) with an operating voltage of 200 kV.

The mechanical properties of the as-cast and as-homogenized samples were measured via a Hysitron TI Premier nanoindenter (Bruker, Billerica, MA, USA) first equipped Berkovich diamond probe. Before testing, the ingots were sliced into 5 mm × 5 mm × 1 mm plates, mechanically ground with silicon carbide paper to 4000-grit, and finally electro-polished for reliable data. The elastic modulus and hardness of the as-cast and as-homogenized samples were obtained by using the load control mode with a peak load of 3000 μN, for which the loading rate was 600 μN/s.

Cylindrical specimens with a diameter of 4 mm and a height of 8 mm for compression tests were machined from as-cast and as-homogenized samples. Compression tests at room temperature with a strain rate of 1 × 10^−4^ s^−1^ were conducted using the Instron 5582 universal testing machine (Norfolk County, MA, USA). To obtain accurate strains and strain rates, the Instron 2601 Linear Variable Differential Transformer (LVDT, Norfolk County, MA, USA) displacement transducer was employed for each specimen.

## 3. Results

### 3.1. Structural Identification of TiZrNbTa Alloys

Figure 1a shows the XRD analysis of the as-cast and as-homogenized TiZrNbTa alloys. It can be seen that the as-cast TiZrNbTa alloy displays a typical BCC crystal structure. However, peaks accompanied by side bands can be observed, especially at high angles, indicating there are two BCC phases (defined as the TiZr-rich BCC1 phase and the NbTa-rich BCC2 phase), with slightly different lattice parameters. Figure 1b shows the enlarged (220) peak profiles and fitting curves of the (220) peak of the as-cast sample for BCC1 and BCC2 by using the Lorentz function. Judging from these fitting curves, the lattice constants of BCC1 and BCC2 phase can be determined to be 0.3375 and 0.3351 nm, respectively, only differing by 0.7%. The lattice constant for each element in the pure metal state is also included in Table 1 for comparison. After homogenization at 1100 °C for 24 h, the as-homogenized alloy seems to be dual BCC structures with lattice constants of 0.3379 and 0.3366 nm, as shown in Figure 1c.

**Figure 2:**
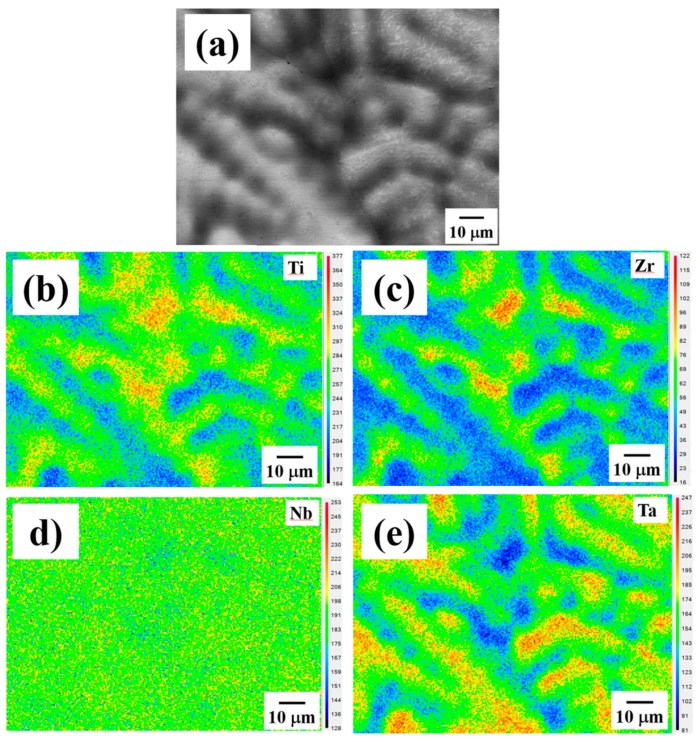
(**a**) The scanning electron microscope (SEM) back-scattered image (BEI) of the as-cast Ti_25_Zr_25_Nb_25_Ta_25_ high-entropy alloy (HEA), and the corresponding composition mapping for (**b**) Ti, (**c**) Zr, (**d**) Nb, and (**e**) Ta. Note that the Nb mapping does not really expose the dendrites (DRs) and inter-dendrites (IDs) clearly. This may be attributed to the fact that the Lα X-ray energy of element Nb (~2.169 eV) is slightly larger than the M absorption edge energy of element Ta (~1.804 eV). Therefore, in the dendrite regions, the Lα X-ray energy of element Nb would be significantly absorbed, resulting in an underestimated amount of the element Nb.

Representative SEM back-scattered images (BEIs) and the corresponding composition mapping are presented in Figure 2. The as-cast TiZrNbTa alloy shown in Figure 2a comprises dendrites (DRs, the brighter region) and inter-dendrites (IDs, the darker region), indicating that the heavier atoms (Nb, Ta) prefer to stay in DRs, while the lighter atoms (Ti, Zr) prefer to locate in IDs. With the help of the mapping results of constituent elements, and consistent with BEI analysis, it can be seen that Ti and Zr are enriched in IDs, as shown in Figure 2b,c, whereas Nb and Ta are enriched in DRs, as shown in Figure 2d,e. With careful examination, no apparent spinodal decomposition microstructure can be seen from SEM (SEI/BEI) images.

However, it should be noted that the Nb mapping by SEM/EDS shown in Figure 2e does not really expose the DRs and IDs clearly. This may be attributed to the fact that the Lα X-ray energy of element Nb (~2.169 eV) is slightly larger than the M absorption edge energy of element Ta (~1.804 eV). Therefore, in the DRs, the Lα X-ray energy of the element Nb would be significantly absorbed, resulting in an amount of the element Nb that is underestimated.

The correct Nb content distribution can be more accurately measured by EPMA, which considers the ZAF correction effect, where Z is the atomic number correction, A is the absorption correction, and F is the fluorescence correction. The chemical compositions and volume fractions of the DRs and IDs in the as-cast TiZrNbTa HEAs measured by EPMA are summarized in Table 2, and are consistent with previous results. The overall observed composition of the as-cast TiZrNbTa HEAs is close to their nominal set value. It should be noted that the Ti content variation in Table 2 is not that pronounced.

The current microstructure and composition distribution are similar to those reported for the as-cast TiZrNbTaMo alloy [16,17]. According to a previous study [16], the microstructure observed in our results can be attributed to the difference in the melting point of the constituent element. During solidification, elements Nb and Ta, with higher melting temperatures, prefer to solidify into primary DRs, while elements Ti and Zr, with a lower melting temperature, are expelled into IDs [16].

**Figure 3:**
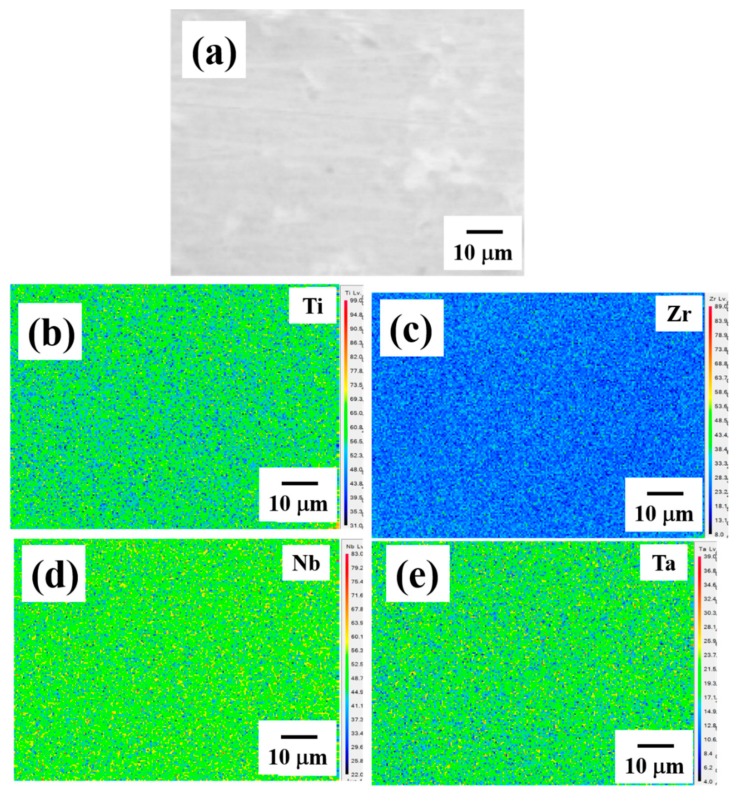
(**a**) The SEM BEI of the as-homogenized Ti_25_Zr_25_Nb_25_Ta_25_ HEA, and the corresponding composition mapping for (**b**) Ti, (**c**) Zr, (**d**) Nb, and (**e**) Ta.

On the other hand, as shown in Figure 3, the BEIs and corresponding composition mapping of the as-homogenized alloy show that the microstructure seems to be a single phase inside the grains, indicating that the DRs and IDs well-dissolve in each other during homogenization. With careful examination through SEM-BEI observation, it can be found that there is no spinodal-like structure inside the grain. However, this result is inconsistent with the XRD results, which exhibit dual BCC structures in the as-homogenized alloy. Therefore, further examinations were conducted by TEM observation.

### 3.2. Mechanical Property Measurement

**Figure 4:**
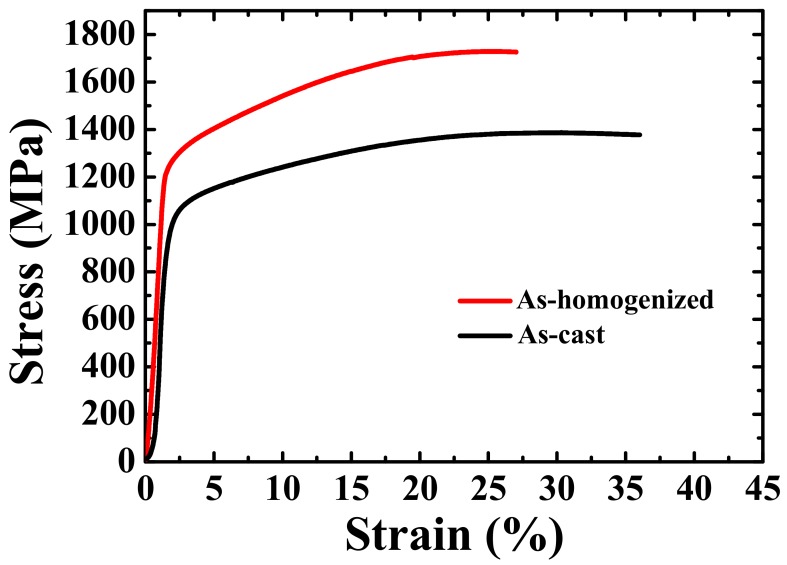
The compressive stress–strain curves of the as-cast and as-homogenized TiZrNbTa alloys.

Figure 4 shows the representative compressive stress–strain curves of the as-cast and as-homogenized TiZrNbTa alloys. Each condition was conducted at least three times. The as-cast TiZrNbTa alloy possesses the compressive yield stress (YS) of 957 MPa and ultimate compressive strength (UCS) of 1389 MPa. After homogenization at 1100 °C for 24 h, the yield stress increases significantly from 957 to 1220 MPa and the UCS increases from 1389 to 1702 MPa. The detailed mechanical properties are listed in Table 3. To further examine the relationship between the microstructure and mechanical properties, nanoindentation was adopted to measure the local hardness from the NbTa-rich phase across to the TiZr-rich phase. In the as-cast sample, there is only a slight difference in the nano-hardness between the two phases in the as-cast TiZrNbTa alloy. The hardness of the NbTa-rich phase is about 5.8 GPa, which is slightly higher than that of the TiZr-rich phase (5.4 GPa), by only 7%. The overall hardness of the as-cast alloy was measured to be 5.6 GPa. After homogenization, the hardness increases from 5.6 to 6.2 GPa, which is consistent with the results of the compression tests. Note that the measured elastic modulus of this new alloy is consistently around 110 GPa, which is much lower than that of equiatomic TiZrNbTaMo HEA (153 GPa) [16] or the commercial Ti-6Al-4V alloy (~135 GPa). This is another benefit of the current new alloy. Based on SEM-BEI observation, the dendritic microstructure of the as-cast alloy was eliminated through high-temperature homogenization and no other phase was observed within the grains. After homogenization, the yield stress and ultimate compressive strength increase significantly with a little sacrifice of plasticity (about 9%), suggesting the formation of some nanoscale phase, which cannot be observed by SEM-BEI. As mentioned above, it is expected that spinodal decomposition would occur in the TiZrNbTa alloy. Traditionally, spinodal decomposition could feature nanoscale interconnected structures [24,26,27] characterized by detailed TEM analysis.

### 3.3. Microstructure Characterization

#### 3.3.1. The As-Cast Samples

**Figure 5:**
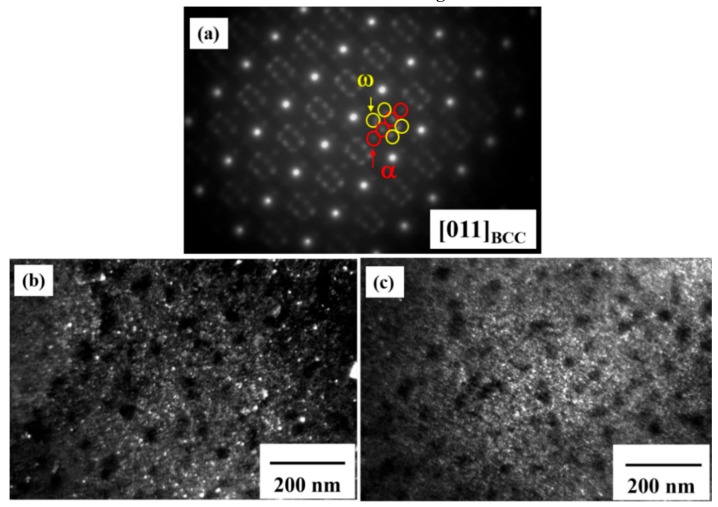
Transmission electron microscopy (TEM) results of the NbTa-rich region in the as-cast alloy: (**a**) selected area diffraction pattern (SADP) of the [011] _BCC_ zone axis; (**b**) dark-field image taken from the α spot, as highlighted by the red arrow in SADP; and (**c**) dark-field image taken from the ω spot, as highlighted by the yellow arrow in SADP.

A selected area diffraction pattern (SADP) with [011] a BCC zone and the corresponding dark-field TEM images taken from the NbTa-rich region of the as-cast TiZrNbTa are presented in Figure 5a–c. The α phase of complex hexagonal structure spots can be observed in the NbTa-rich phase of the as-cast alloy. The dark-field image obtained from the α spot shows that α precipitate has a size of about 7 ± 2 nm, distributed evenly in the NbTa-rich matrix in Figure 5b. Furthermore, another fine precipitate formed, which was the ω phase with an ordered hexagonal structure. This ω precipitate is a metastable phase with lattice constants of a = 0.4739 nm and c = 0.2902 nm, related to the BCC matrix (a=√2.a_BCC_, c= (√3/2) a_BCC_) and commonly found in Ti-based alloys, and is also observed to spread uniformly in the NbTa-rich matrix, as shown in Figure 5c. The size of this ω precipitate was measured to be about 5 ± 1 nm. Note that both α and ω fine precipitates were found to only be present in the NbTa-rich DR regions, and not in the TiZr-rich ID region.

#### 3.3.2. The As-Homogenized Samples

**Figure 6:**
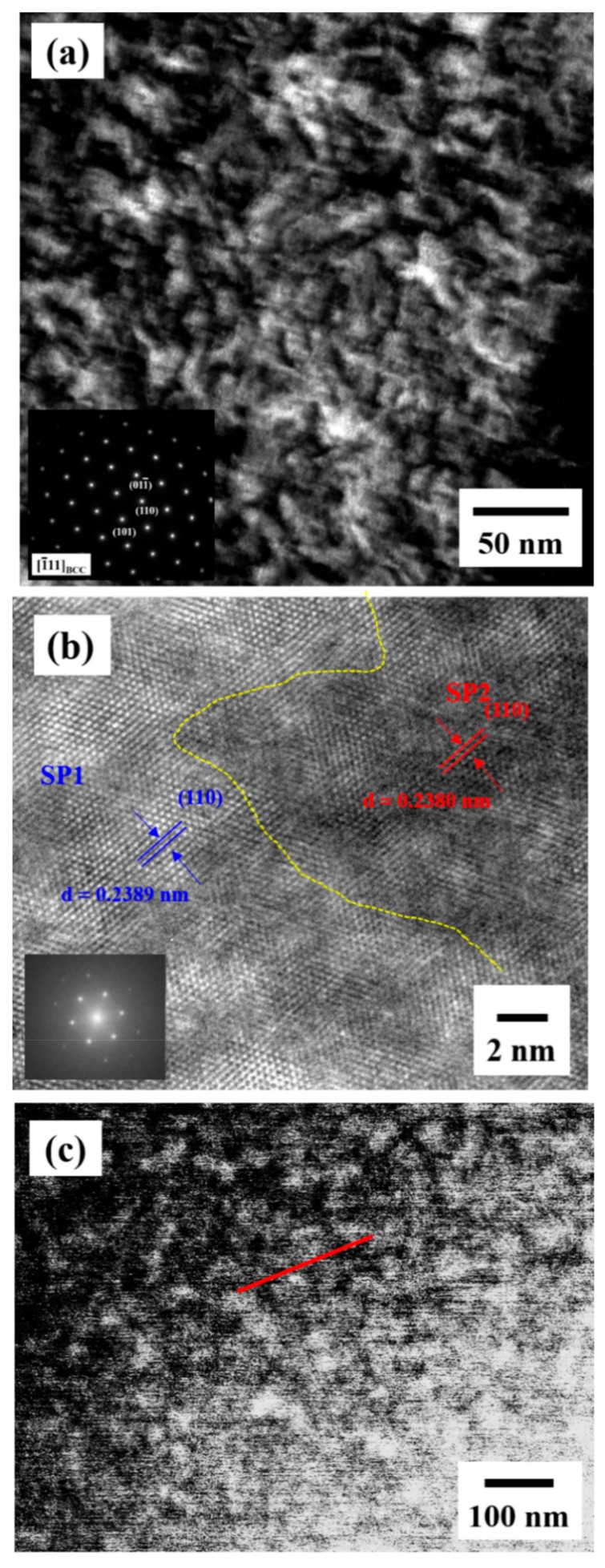
(**a**) The TEM dark-field images, (**b**) the corresponding high-resolution TEM images, and (**c**) the HAADF-STEM image of the interconnected structure in the as-homogenized alloy.

**Figure 7:**
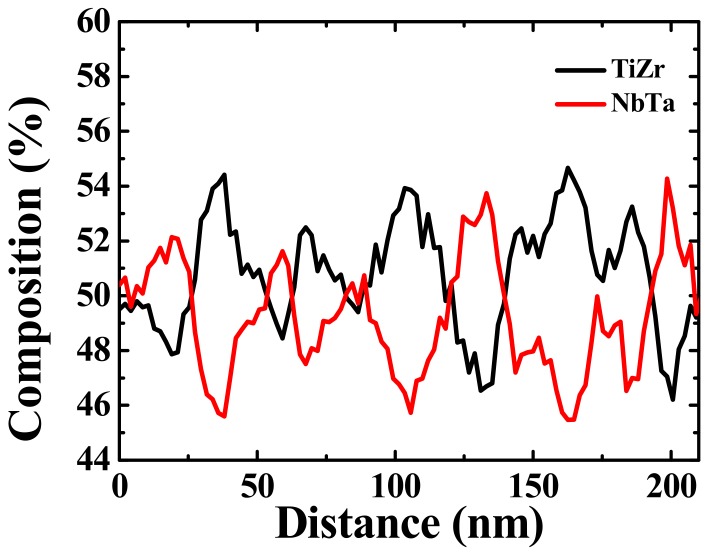
STEM-EDS line scan result of the spinodal interconnected structure along the red line of Figure 6c in the as-homogenized alloy.

**Figure 8:**
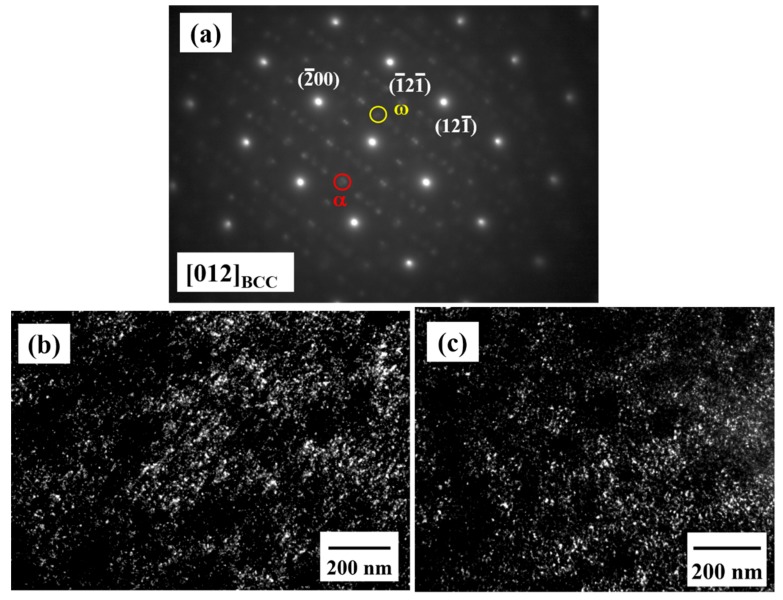
TEM results of the α and ω precipitates in the as-homogenized alloy: (**a**) SADP of the [012] BCC zone axis; (**b**) dark-field image taken from the α spot, as highlighted by the red circle in SADP; and (**c**) dark-field image taken from the ω spot, as highlighted by the yellow circle in SADP.

In order to characterize the structure further, TEM observation was carried out and the results are shown in Figure 6. The TEM dark-field image (Figure 6a) reveals a dual phase interconnected structure with a wavelength of about 20 nm inside the grains. Note that the inserted SADP shows that the as-homogenized alloy exhibits a BCC structure. Figure 6b presents the high-resolution TEM image and related fast Fourier transform (FFT) of the dual BCC phase interconnected structure. The (110) d-spacing of the spinodal region 1 (SP1) and spinodal region 2 (SP2) was measured to be 0.2389 and 0.2380 nm, respectively. As a result, the lattice mismatch between SP1 and SP2 is extremely small, demonstrating a coherent interface between them. A high-angle annular dark-field scanning transmission electron microscope (HAADF-STEM) image, as shown in Figure 6c, demonstrates that the spinodal structure consists of a dual phase with different Z-contrasts. The bright contrast region is the phase with a higher atomic number, Z, and the dark contrast region is the phase with a lower Z. Furthermore, the scanning transmission electron microscope energy dispersive X-ray spectroscopy (STEM-EDS) line scan was applied along the red line labeled in Figure 6c. The STEM-EDS line scan result of the spinodal structure is presented in Figure 7. Since the heat of mixing between Ti and Zr, as well as Nb and Ta, is 0 kJ/mol, these four elements were divided into a TiZr group and an NbTa group to present the apparent compositional fluctuation for the spinodal structure. TiZr is enriched in SP1 (dark contrast in Figure 6c), while NbTa is enriched in SP2 (bright contrast in Figure 6c). This may be attributed to the positive heat of mixing between TiNb, TiTa, ZrNb, and ZrTa, as listed in Table 1. The wavelength of the spinodal structure is about 20 nm on average, which is consistent with the results obtained by TEM observation. As aforementioned, with the homogenization process at 1100 °C, the DRs and IDs in the as-cast sample were well-dissolved in each other. During fast water quenching, the nano-scaled spinodal structure forms [28,29]. The spinodal decomposition spontaneously occurs and the diffusion process for coarsening will be limited by fast cooling [28]. Moreover, a spinodal structure with a wavelength of about 40–60 nm [30,31], which is very close to our results (about 20 nm), strengthens the alloys effectively, indicating the reason for such a good performance for the as-homogenized sample. In addition, it should be noted that both α and ω nano-precipitates can be observed within the grains, as shown in Figure 8. These two precipitates maintain a size that is similar to that in the as-cast alloy and distribute evenly within the grains, revealing that the precipitates are formed during the quenching [32]. Combining the mechanical property measurements and TEM observation, the spinodal interconnected structure is responsible for the strength increment.

## 4. Discussion

### Relationship between Spinodal Decomposition and the Mechanical Response

Based on the compression testing, Vickers hardness measurements, and nanoindentation, it was revealed there is a consistent trend in that the homogenization process on the TiZrNbTa alloy could significantly improve the mechanical properties (about 27% for YS). This finding is due to the fine spinodal structure, which is generally a good strengthening mechanism, as reported in the literature [25,33,34]. It is believed that there are multiple strengthening mechanisms in this alloy, such as (i) grain size strengthening, (ii) solid solution strengthening, (iii) precipitation strengthening by α and ω phase, and (iv) spinodal decomposition strengthening. Since the grain size (about 62.1 μm) and solid solution strengthening contributions in the as-cast and as-homogenized samples are not as great as the other two strengthening effects, an attempt has been made to evaluate the contribution of precipitation and spinodal decomposition strengthening.

Firstly, both the α and ω precipitates are considered to be obstacles to dislocation movement and could strengthen the material effectively. Since these two precipitates are both large enough and could not be sheared through, they could contribute to strengthening by the Orowan mechanism [35,36,37]:

(1) $$\tau_{c}=\frac{0.8\times 2G\Gamma }{b\;(\mathrm{Ls}-2\mathrm{rs})}=\frac{0.8\;\mathrm{Gb}}{\;2\pi \;(\mathrm{Ls}-2\mathrm{rs})}\ln\left(\frac{\mathrm{rs}}{b}\right),$$

where b is the Burger’s vector and Γ is the dislocation line tension, given by

(2) $$\Gamma =\frac{{\mathrm{Gb}}^{2}}{4\pi }\ln\left(\frac{\mathrm{rs}}{b}\right),$$

with the shear modulus G being equal to

(3) $$G=\frac{E}{2(1+\nu )},$$

where υ is the Poisson’s ratio, ~1/3. The square lattice spacing L_s_ is expressed as

(4) $$L_{s}=(\frac{2\pi }{3f}{)}^{\frac{1}{2}}<r>,$$

where <r> is the average radius and f is the volume fraction of precipitates. For the calculation of f, while both the overlapping and truncation effect are considered, the volume fraction, f, is given by [38]

(5) $$f=\frac{-4<r>\ln(1-A_{p})}{2<r>+3h},$$

where *A_p_* is the projection area fraction estimated from TEM observation and h is the TEM foil thickness. Finally, the planar radius is equal to

(6) $$r_{s}=\frac{\pi \;<r>}{4},$$

By inputting the related measured values into these parameters, as summarized in Table 4, the strengthening contributions provided by the α and ω particles can be evaluated. From numerous TEM micrographs, the volume fraction of the α and ω particles inside the NbTa-rich domains has been determined to be 7.1% and 4.1%, respectively, in the as-cast samples. By incorporating the dislocation line tension and particle spacing into Equation (1), the critical resolved shear stress strengthening contribution of these two particles has been estimated to be 169 and 146 MPa, respectively, as listed in Table 4. By assuming that the Taylor factor M is 3, the strengthening to the normal yield stress, σ_y_, can be determined, namely,

σ_y_ = M τ_c_ = 3τ_c_, (7)

Therefore, σ_y_ has been calculated to be 507 and 438 MPa, respectively, for the strengthening to the yield stress by the σ and ω particles.

By assuming the simple linear addition rule [39,40], the overall contribution of these particles is thus 945 MPa. However, since the σ and ω particles are only seen in the NbTa-rich domains, which occupy about 54% of the volume fraction (Table 2), the overall strengthening increment to the as-cast sample has been estimated to be 510 MPa. In addition, there should be some strengthening contribution from the TiZr-rich domains, and this part has been estimated to be about 475 MPa, judging from the hardness measurement from the NbTa-rich and TiZr-rich domains in Table 3, namely, 510 × (5.4/5.8) ~475 MPa. By combining these two contributions, the overall strengthening gives 510 + 475 = 985 MPa, as listed in Table 4. In fact, this is not far from the measured yield stress of 957 MPa for the as-cast sample.

After homogenization, the strengthening increment should be mostly attributed to the spinodal decomposition strengthening, which has been proposed to be given by [41]:

(8) $$\tau_{c}=0.122(A\eta Y{)}^{\frac{5}{3}}{\left(\frac{\lambda b}{\Gamma }\right)}^{\frac{2}{3}},$$

where *A* is the amplitude of composition modulation, λ is the wavelength of that modulation, *η* is related to the variation of the lattice constant with composition, and Y is related to the elastic constant. All the values for the related parameters for the as-homogenized alloy, listed in Table 4, can be employed to conduct an estimation of the critical resolved shear stress, which was determined to be 79 MPa. The yield stress contributed by the spinodal decomposition for the as-homogenized sample was 237 MPa. In addition, based on numerous TEM observations, the size and distribution of α and ω precipitates in the as-homogenized sample are basically the same as those in the as-cast one. Therefore, there should be not much difference in the volume fraction of α and ω precipitates (~7.1% and ~4.1%, respectively) between the as-cast and the as-homogenized samples. Besides, since the α and ω particles can be observed everywhere in the as-homogenized samples, it is assumed that the strengthening contribution of α and ω would give 507 + 438 = 945 MPa. The overall strengthening, subject to α and ω precipitation hardening and spinodal decomposition strengthening, is 945 + 237 = 1182 MPa, close to the measured yield stress of 1220 MPa.

## 5. Conclusions

As elaborated in this paper, the effects of precipitation and spinodal decomposition strengthening on the mechanical properties of the TiZrNbTa high-entropy alloy have been demonstrated. The main findings can be drawn as follows:

1. The as-cast TiZrNbTa is composed of dual BCC phases, namely, the TiZr-rich inter-dendrite domains (IDs, BCC1) and the NbTa-rich dendrite domains (DRs, BCC2). The lattice constant of BCC1 and BCC2 has been determined to be 0.3375 and 0.3351 nm, respectively. By TEM examinations, α and ω precipitates measuring about 7 and 5 nm, respectively, have been found to be distributed uniformly in the NbTa-rich domains;
2. In the as-cast TiZrNbTa alloy, as verified by nanoindentation tests, the NbTa-rich phase, residing within the grains, possesses a higher hardness of 5.8 GPa than that of the TiZr-rich phase (5.4 GPa). The as-cast TiZrNbTa alloy exhibits a compressive yield stress (YS) of 957 MPa and ultimate compressive strength (UCS) of 1389 MPa, with a failure plastic strain of 36%. The strengthening was rationalized to have occurred via Orowan strengthening by the α and ω precipitates;
3. After the alloy was homogenized at 1100 °C for 24 h, the DRs and IDs well-dissolved in each other. Furthermore, spinodal decomposition occurred during the quenching and the interconnected structure with a wavelength of about 20 nm has been characterized to be composed of TiZr-rich and NbTa-rich phases, both of which have a BCC structure. In addition, during the quenching, the α and ω particles also precipitated within the grains uniformly;
4. The as-homogenized TiZrNbTa alloy possesses a compressive yield stress of 1220 MPa, which is 27% higher than that of the as-cast alloy. Moreover, after homogenization, the UCS increased from 1389 to 1702 MPa with a failure plastic strain of 27%. The increment of strength has been attributed to the formation of a spinodal interconnected structure.

## Figures and Tables

**Table 1:** Heat of mixing between constituent elements and the lattice constant, a, in their pure metal state, and their related physical properties.

	Ti	Zr	Nb	Ta
Ti	--	0	2	1
Zr	0	--	4	3
Nb	2	4	--	0
Ta	1	3	0	--
a (nm)	0.295/0.469	0.323/0.515	0.330	0.330
Modulus E (GPa)	116	67	105	186

**Table 2:** Emission electron probe micro-analyzer (EPMA) results of the as-cast Ti_25_Zr_25_Nb_25_Ta_25_ high-entropy alloy. Note that DR and ID refer to the dendrite and inter-dendrite region, respectively.

Alloy	Region	Structure	Ti	Zr	Nb	Ta	Volume Fraction (%)
TiZrNbTa	Overall	BCC	24 ± 1	26 ± 1	26 ± 1	24 ± 1	100
	DR		22 ± 2	19 ± 2	29 ± 1	30 ± 2	54
	ID		26 ± 1	35 ± 2	22 ± 1	17 ± 2	46

**Table 3:** Results of the Ti_25_Zr_25_Nb_25_Ta_25_ alloy, obtained from the microhardness (Hv), nanoindentation (E and H), and compression testing (yield stress (YS), ultimate compressive strength (UCS), and failure compression strain e%) under the as-cast and as-homogenized conditions.

Condition	Region	Hv	E (GPa)	H (GPa)	YS (MPa)	UCS (MPa)	e%
	Overall	380	111 ± 6	5.6 ± 0.4	957 ± 25	1389 ± 5	36 ± 1
As cast	DR	--	116 ± 4	5.8 ± 0.3	--	--	--
	ID	--	106 ± 3	5.4 ± 0.3	--	--	--
	Overall	440	109 ± 4	6.2 ± 0.2	1220 ± 19	1702 ± 20	27 ± 1
As homogenized	DR	--	--	--	--	--	--
	ID	--	--	--	--	--	--

**Table 4:** Summary of the inserted data and the calculated strengthening contributions. Note that SD represents spinodal decomposition.

Parameter	Datum	Parameter	Datum	Parameter	Datum
<r> for α	~7 nm	<r> for ω	~5 nm	h	65 nm
Ap for α	41%	A_p_ for ω	34%	E	111 GPa
f for α	7.1%	f for ω	4.1%	G	42 GPa
Ls for α	27.0 nm	Ls for ω	34.5 nm	λ	~20 nm
A	~3 at%	η	~0.058	Y	~115 GPa
τ_c_ for α	169	τ_c_ for ω	146	τ_c_ for SD	79
σ_y_ for α	507	σ_y_ for ω	438	σ_y_ for SD	237

σ_y_ for (α + ω) in NbTa-rich DRs (54% in volume fraction) (507 + 438) × 0.54 = 510 MPa; σ_y_ for the TiZr-rich IDs (46% in volume fraction) ~475 MPa; σ_y_ for spinodal decomposition ~237 MPa; Total σ_y_ for the as-cast sample ~985 MPa; Total σ_y_ for the as-homogenized sample ~945 + 237 = 1182 MPa.
